# A Large Outbreak of Gastrointestinal Infections Associated with *Escherichia coli* and Consumption of Milk Served in 25 schools in Toyama, Japan, June 2021

**DOI:** 10.14252/foodsafetyfscj.D-25-00027

**Published:** 2026-09-25

**Authors:** Katsumi Mizukami, Noriko Noguchi, Yuichiro Yahata, Yuuki Tsuchihashi, Kaoru Kurosaki, Kenji Ohya, Shouhei Hirose, Tomoya Yoshinari, Takahiro Ohnishi, Kenichi Lee, Sunao Iyoda, Hideko Kusunoki, Keiko Tsukada, Natsuki Yokota, Akane Kiritani, Mayumi Eino, Kazutaka Nakada, Akina Raijou, Yuichiro Hyakkoku, Takeo Edo, Tomikatu Suzuki, Kenji Takinami, Yukihiro Akeda, Yukiko Hara-Kudo, Tomimasa Sunagawa

**Affiliations:** 1 Toyama City Public Health Center, 459-1 Ninagawa, Toyama-shi, Toyama 939-8222, Japan; 2 National Institute of Infectious Diseases, 1-23-1 Toyama, Shinjuku-ku, Tokyo 162-8640, Japan; 3 National Institute of Public Health, 2-3-6 Minami, Wako-shi, Saitama 351-0197 Japan; 4 National Institute of Health Sciences, 3-25-26 Tonomachi, Kawasaki-ku, Kawasaki City, Kanagawa 210-9501, Japan; 5 Hoshi University, 2-4-41 Ebara, Shinagawa-ku, Tokyo 142-8501, Japan.

**Keywords:** large outbreak, school lunch, *Escherichia coli*, HACCP, foodborne disease, small-scale milk processing company

## Abstract

Severe foodborne disease outbreaks occurring in the school setting are a major public health issue. On June 17, 2021, Toyama City Public Health Center received a report of a suspected outbreak of food poisoning from the Board of Education of Toyama City. The board reported that 459 students and 5 teachers were absent from school or left early on June 17. We investigated the suspected outbreak and examined the implementation of control measures in a small-scale milk processing company linked to the outbreak. In particular, we performed an epidemiological investigation involving a traceback and traceforward investigation, an inspection of the processing company, a microbiological analysis, a hazard analysis, and an evaluation of critical control points. In total, 1,896 individuals developed at least one gastrointestinal symptom and were absent from school and/or left early. In total, 25 schools and childcare facilities were affected. The epidemiological investigation showed that consumption of school lunch milk from a specific milk company was significantly associated with absence from school on June 16–18, with odds ratios ranging from 2.2 to 21.7. No cases were linked to other distribution facilities. Milk and stool samples yielded *Escherichia coli* of undetermined pathotype. The milk processing facility had 20 hazards, including the heat treatment machine. The facility did not undergo routine checks and maintenance. The temperature detector sensor was manufactured to have 20 copper wires, but just 2 wires were intact, leading to a possible overestimation of temperature. We recommended that the facility routinely measure direct temperature and implement checks and maintenance of the heat treatment unit.

## Introduction

Foodborne disease outbreaks may cause severe illness and even death, particularly in children, the elderly, pregnant women, and people with underlying health conditions or weakened immune systems^1^^)^. Outbreak prevention is one of the most important public health issues worldwide. The Codex Alimentarius (CODEX) describes the general principles of food hygiene and identifies the essential components of food hygiene applicable throughout the food chain to help ensure that food is safe and suitable for human consumption^2^^)^. The report recommended the application of a hazard analysis and critical control point (HACCP)-based approach as a means to enhance food safety.

Since June 1, 2021, the Japanese Ministry of Health, Labour and Welfare (MHLW) has implemented a HACCP plan for food hygiene control in all food processing companies in Japan^3^^)^. One of the institutional food service facilities based on HACCP in Japan is the public-school lunch overseen by the Board of Education of Toyama City, which has been linked to several large and severe outbreaks in schools in Japan^4^^,^^5^^)^. Although non-pasteurized dairy products can be contaminated by pathogens in the dairy environment, these microbes would be eliminated by proper pasteurization^6^^)^. In particular, raw milk may be contaminated with numerous types of microbiological agents^7^^,^^8^^,^^9^^)^, which can be destroyed via pasteurization^10^^)^. The CODEX recommends that milk producers apply the HACCP system in order to minimize their presence in the raw material^11^^)^.

### Outbreak detection

On June 17, 2021, the Toyama City Public Health Center (TCPHC) in Japan received information on a suspected outbreak of food poisoning from the Board of Education of Toyama City. The Board of Education reported that, as of June 17, 464 students from 18 schools had been absent or had left school early due to diarrhea, abdominal pain, or fever. Preliminary information suggested that the school lunch was the only meal or event identified in common with the ill students. All cases consumed milk produced by milk-processing company T (hereafter, company T). The company issued a voluntary recall of the milk on June 17 based on preliminary exposure information, before formal epidemiological analysis. TCPHC then initiated an investigation for prevention and control purposes. On June 28, TCPHC requested that the National Institute of Infectious Diseases (NIID) conduct an outbreak investigation and implement control measures at a small-scale milk-processing company.

Here, we describe the details of the epidemiological investigation and recommend methods for remediation.

## Materials and Methods

We conducted eight types of investigation: a descriptive epidemiological investigation, a review of school lunch menus for 25 schools, a menu-based cohort study for two schools, a cross-sectional study of 25 schools, a traceback and traceforward investigation, inspection company T, microbiological testing, and hazard analysis and evaluation of critical control points (CCPs).

TCPHC conducted an epidemiological investigation per the Japanese Food Sanitation Act^12^^)^. The public health center collected epidemiological information on the symptomatic cases of students and teachers, including the school name, school grade, symptoms, date and time of onset, and the school lunch consumed. In addition, TCPHC collected leftover milk from company T and preserved the school lunch served from June 14 to 18 in the 25 schools reporting cases.

### Descriptive analysis of the outbreak

In this investigation, we defined cases as a student or staff member, including teachers, administrative staff, and any other staff belonging to a nursery school, kindergarten, elementary school, or other type of school, who developed at least one self-reported gastrointestinal symptom such as abdominal pain, diarrhea, and/or fever (≥38.0°C) in Toyama City from June 14 to 18, 2021, and met the following criteria:

• Confirmed case: laboratory confirmation of *E. coli* with OgGP9^13^^)^ (O-genotyping)

• Suspected case: without laboratory confirmation of *E. coli*

### Menu-based cohort study

We conducted a menu-based retrospective cohort study of the school lunch served to students in the fourth to sixth grades at two schools. A questionnaire survey of 302 students was conducted on June 7–18. We excluded cases with missing data on school lunch consumption and/or with an onset before consumption of the school lunch on June 14. We calculated the risk ratio (RR) and 95% confidence interval (95%CI) for the risk of consuming food items from the school lunch each day.

### Cross-sectional study

We conducted a cross-sectional study to evaluate the relationship between exposure, defined as whether the milk was supplied by company T, and absence from school from June 14 to 16, 2021, using school absence data from 1,888 students. Students in schools receiving company T milk were classified as exposed, and those in schools receiving milk from other companies as unexposed.

We calculated odds ratios (ORs) and 95%CIs for the association between company T milk receipt and absence.

### Traceback and traceforward investigation

Traceback and traceforward investigations were conducted for each food material in the school kitchen by TCPHC. The public health center collected invoices of all food materials and records of the cooking process. In addition, TCPHC conducted a traceforward investigation of the milk produced by company T.

### Inspection of the milk-processing company

TCPHC inspected company T four times between June 17 and July 5, 2021. On July 2, TCPHC reviewed the company with NIID. The inspection team checked the daily routine followed for milk processing and interviewed the chief executive officer about this procedure. In addition, the team collected the milk-processing records on the day. Finally, TCPHC collected processing records, logistics records, and invoices for purchased milk.

### Laboratory testing

TCPHC analyzed stool samples collected from 64 cases for the presence of microbiological agents and/or toxins. Stool samples were collected on June 17–21 while TCPHC collected 10 days of preserved lunches, including milk, from the 25 schools and from milk-processing equipment such as raw milk storage tanks, strainers, storage tanks, and milk-filling and packing machines on June 18. TCPHC isolated stool and food samples for the detection of *Staphylococcus aureus*, *Clostridium perfringens*, *Bacillus cereus*, *E. coli*, *Shigella*, *Salmonella*, *Vibrio parahaemolyticus*, *Campylobacter* spp., *Yersinia*, *Aeromonadaceae*, *Plesiomonas*, and *Norovirus*. Tested virulence markers, including *stx1*, *stx2*, and *eae*, were not detected in the *E. coli* isolates.

### Hazard analysis and evaluation of CCPs

In accordance with the Japanese Food Sanitation Act, we conducted a hazard analysis of company T. TCPHC inspected the entire milk production process. TCPHC checked each part of the milk processing plant while TCPHC and NIID performed the hazard analysis and evaluated the CCPs for each item in the plant.

## Results

### Characteristics of cases

**Fig. 1.**
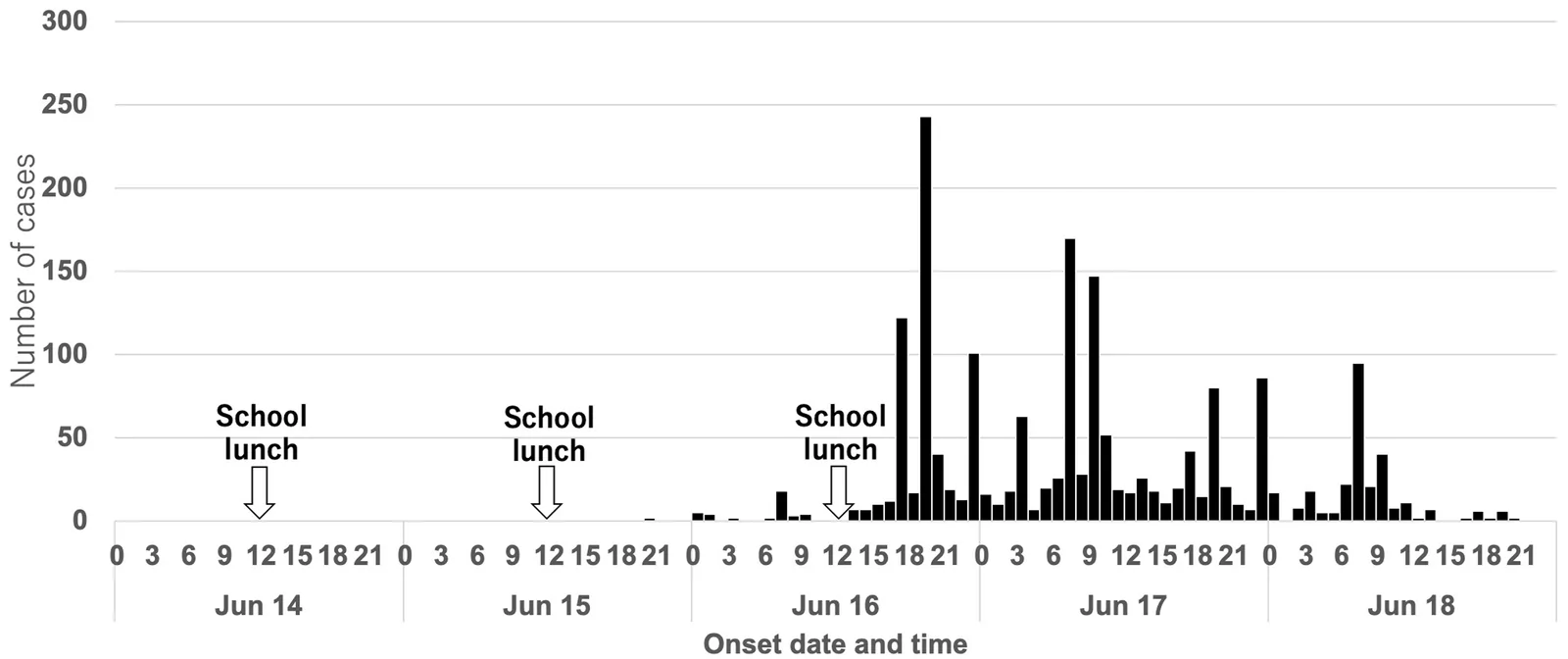
Epidemic curve of the gastrointestinal illness outbreak in Toyama in 25 schools in June 2021 (N=1,850)

The total number of individuals who developed gastrointestinal symptoms was 1,896 from 25 schools as of July 25, 2021, and we were able to collect the epidemiological data of 1,850 cases. The first case showed symptoms at 18:00 on June 14 (**Figure 1**). In total, 25 institutions reported cases, including elementary schools (1,097 cases), junior high schools (481 cases), and kindergartens and nursery schools (318 cases) (**Supplementary Table 1**). We were able to collect the epidemiological data of 6,243 students, teachers, and administrative staff from the schools. Of the 6,243 people who consumed school lunch at the 25 facilities, 1,850 (30%) developed gastrointestinal illness. Of these 1,850 cases, 1,472 (80%) developed abdominal pain and 1,006 (54%) developed diarrhea (**SupplementaryTable 2**). Abdominal pain was reported by 78% of individuals aged 5–9 years (626 of 802) and by 88% of those aged 10–14 years (786 of 894). Diarrhea developed in 60% of individuals aged 10–14 years (538 of 894) and in 48% of those aged 5–9 years (386 of 802). Finally, 43% of those aged 1–4 years developed fever (49 of 113).

### Risk of consumption of school lunch milk: a menu-based cohort study

Of the 302 students in two elementary schools (school 1: 208 students; school 2: 94 students) who were investigated in a menu-based cohort study, 91%–99% consumed school lunch milk during June 14–16 (Supplementary **Table 4**). About 90% consumed food items other than milk. The overall attack rate (AR) of gastrointestinal illness was 29% (88 of 302) (**Table 1**). Of students who consumed milk, the AR of gastrointestinal illness was 30% (86 of 286). Of those who did not consume milk, the AR of gastrointestinal illness was 13% (2 of 16). Thus, the overall RR was 2.4 (95%CI: 0.7–8.9). The daily RRs for school lunch milk consumption were 2.4 (95%CI: 0.7–9.0) on June 14, 2.5 (95%CI: 0.7–9.1) on June 15, and 0.9 (95%CI: 0.3–2.6) on June 16 (**Table 1**). Our analysis revealed that 82 cases (92%; **SupplementaryTable 3**) consumed the milk three times.

**Table 1.** Menu-based cohort study of the risk ratio for milk consumption on different dates during the gastrointestinal illness outbreak in Toyama in June 2021 (n=302)

Consumed milk	Onset		Risk	RR ^a)^	95%CI ^b)^		
	Ill	Not ill					
Overall ^c)^							
Consumed	86	200	0.30	2.4	0.7	–	8.9
Not consumed	2	14	0.13				
Total	88	214	0.29				
June 14							
Consumed	85	196	0.30	2.4	0.7	–	9.0
Not consumed	2	14	0.13				
Total (missing=4) ^d)^	87	211	0.30				
June 15							
Consumed	87	197	0.31	2.5	0.7	–	9.1
Not consumed	2	14	0.13				
Total (missing=2) ^c)^	89	211	0.30				
June 16							
Consumed	27	193	0.12	0.9	0.3	–	2.6
Not consumed	3	18	0.14				
Total (missing=3) ^c)^	30	211	0.12				

a) RR: Relative Risk

b) 95%CI: Confidence Interval

c) School milk consumption on at least 1 day in June 14–16

d) Missing data: excluded a case of onset before June 16

**Table 2.** Analysis of the critical control points in company T

No.	Equipment	Sub-equipment	Check item	Result for infection	Possibility of hazard
1	Raw milk tank		Washing of inside of raw milk tank	Insufficient use (No CIP washing)	Yes
2	Joint of raw milk tank		Washing of joint	No hand washing or CIP washing	Not denied
3	Strainer		Washing of strainer	Without CIP washing	Yes
	Balance tank		CIP washing	Sufficient washing	No
	Strainer		CIP washing	Sufficient washing	No
	Storage tank		CIP washing	Sufficient washing	No
4	Homogenizer		Pressure gauge	Trouble/insufficient function	Not denied
5	Heat treatment	Washing	Washing the inside of the heat-treatment system	CIP washing	No
		Heat treatment	Heat treatment	Insufficient measurement of temperature	Yes
		Thermometer	Inspection and maintenance	Insufficient electrical line/insufficient function and insufficient record of temperature	Yes
		Flow diversion valve	Inspection and maintenance	Insufficient function, inspection, and maintenance	Yes
		Measurement of flow	Measurement of flow of milk	No continuous recording	Not denied
			Actual flow of milk	Indirect measurement	Yes
		Joint of strainer	Washing the inside of the joint	Inspection not performed	Yes
6	Surge tank	Tank	Ventilation	Contact with outside air	Yes
		Flow	Contamination from the environment	Overflow of milk	Not denied
		Spray ball	Contamination from the environment	Spray ball coming into contact with the milk	Not denied
7	Filling machine		Inspection and maintenance	Insufficient inspection and maintenance/washing	Not denied
		Packing machine	Handling and sanitization	Insufficient preservation of packaging and recording of lot numbers	Not denied
5–7	Joint of strainer		Mechanical seal	Partial leakage of milk	Not denied
1–8	Standardized personal protective equipment	Hand hygiene	Hand sanitizer and disposable gloves	Insufficient hand washing and glove changes	Yes
		Rubber boots	Sanitization	No sanitization of rubber boots	Yes
		Chemical disinfectant	Preserving	Insufficient preservation of sanitizer	Not denied
9	Refrigerator		Power outrage/Loss of power	No use of UPS	Low
			Storage temperature	No records	Not denied

### Association of consumption of the milk supplied by company T and absence from school or early departure due to sickness: a cross-sectional study

Being enrolled in a school that received milk from company T was significantly associated with being absent from school or leaving school early on June 16 (OR=2.2, 95%CI: 1.4–3.5), June 17 (OR=12.1, 95%CI: 8.6–16.8), and June 18 (OR=21.7, 95%CI: 15.8–30.0) (**Supplementary Figure**). The recorded consumption proportion for school lunch milk ranged from 93% to 100% (**Supplementary Table 4**).

### Traceback investigation and traceforward investigation

**Fig. 2.**
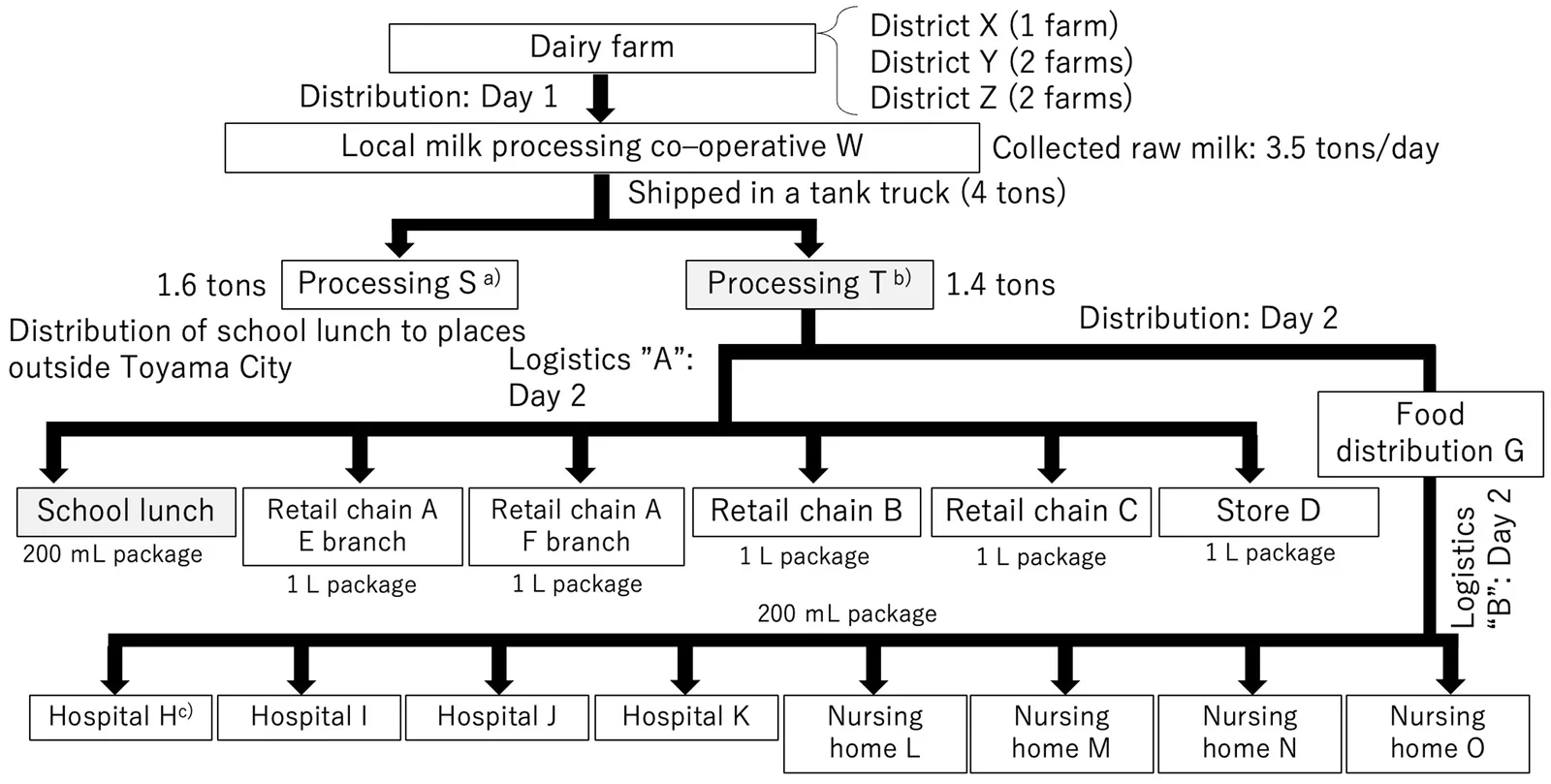
Traceback and traceforward for milk

TCPHC conducted a traceback investigation for company T (**Fig. 2.**). Company T was supplied by the local co-operative federation of milk processors, which itself collected from five dairy farms in three districts. The total amount of milk obtained from the five dairy farms was about 3.5 tons/day. The co-operative supplied milk to milk-processing company S (hereafter, company S) and company T. Company S received 1.6 tons/day from the co-operative while company T received 1.4 tons/day. Company T processed 6,773 200-ml packs of milk on June 14 and 16 and 6,723 packs on June 15. Regarding the distribution of the 200-ml milk pack to the 25 schools, 6,306 packs were distributed on June 14 (93%), 6,306 packs on June 15 (94%), and 6,306 packs on June 16 (93%). The company processed all 200-ml milk packs as a single batch. Company S supplied milk to schools of municipalities other than Toyama City. Company S had no reports of health problems from consumers or school students. Company T supplied the same batch of milk to 26 schools (25 schools: 200 mL; one school: 500 mL), four supermarkets (500 mL or 1 L), four hospitals (200 mL), four nursing homes (200 mL), and a grocery store (1 L). No health problems were reported for milk from hospitals, nursing homes, or retail stores based on active case finding. No other ingredients were common to the schools

### Detection of microbiological agents

Samples were collected from 64 cases who developed at least one gastrointestinal symptom, such as diarrhea or abdominal pain. In the 64 stool samples, the agents detected were *E. coli* (62 cases), *Staphylococcus aureus* with enterotoxin (1 case), *Clostridium perfringens* (1 case), and *Norovirus* GII (1 case). We did not isolate major pathogenic *E. coli* strains from the stool samples by culture ^13^^)^. Other agents were not isolated and/or detected from the stool samples. Regarding milk samples, we were able to contact 28 institutions, which included 12 elementary schools, 5 nursery schools, 3 high schools, 2 kindergartens, 2 junior high schools, 2 other educational institutions, 1 certified childcare center, and 1 milk processing company. Of the 28 institutions, we collected 19 milk samples, and we isolated *E. coli* from 11 of the 19 milk samples ^13^^)^. Of the 11 milk samples, 3 were served with school lunch on June 14–16 in Elementary School 1. No major virulence genes associated with established diarrheagenic *E. coli* were detected in the milk samples ^13^^)^. Except for the raw milk storage tank, all milk processing equipment samples were negative for *E. coli* and the other tested agents. Enterotoxin-producing *B. cereus* was found in the raw milk storage tank.

### Management of control measures

On June 17, company T conducted a voluntary recall of the milk distributed on June 14–16 that comprised all unconsumed milk from schools or other institutions and any milk being sold in stores. The company temporarily closed on June 18. In accordance with the Food Sanitation Act, the company was temporarily banned from producing milk by TCPHC on June 19. On August 2, the processing company resumed the production of dairy items.

### Inspection and hazard analysis of company T

**Fig. 3.**
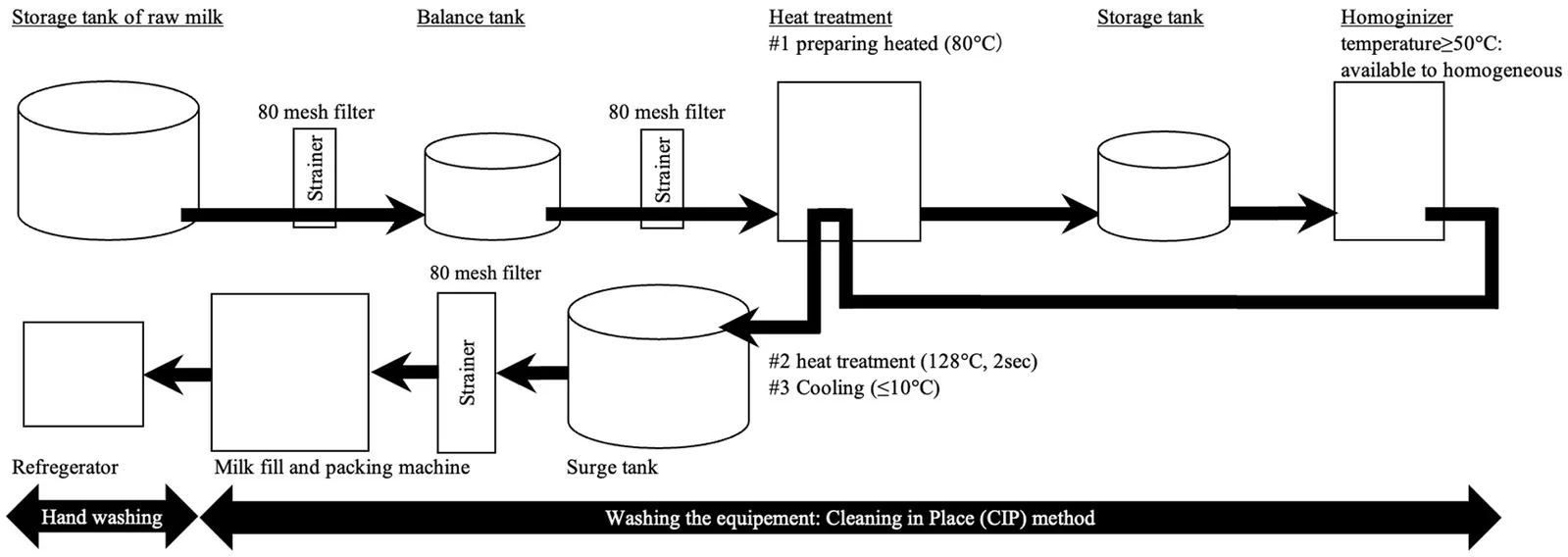
Processing of the milk in milk processing company T

TCPHC conducted an inspection of company T from June 17 to July 23, 2021. Several hazards were found in relation to the milk processing procedure (**Fig. 3.**). The storage tank was washed by hand by a staff member wearing insufficiently sanitized rubber boots. The facility implemented the cleaning-in-place washing process from the balance tank to the milk-filling machine but not in the storage tank for raw milk or in the strainer before the balance tank. Regarding the homogenizer, the pressure measurement was inappropriate for the device. Moreover, the homogenizer did not undergo routine inspection or maintenance by the company. TCPHC conducted a routine inspection of the milk processing facility once a year and tested milk samples every 2 months. TCPHC conducted a regular inspection of 26 items based on the checklist (**Supplementary Table 5**).

The heat-treatment machine contained a resistance temperature detector (RTD) sensor for measuring temperature, which does not directly measure temperature but rather electrical resistance as a reflection of the heat-treatment temperature. The RTD sensor was designed with 20 copper wires for measuring temperature; however, only 2 were still intact at the inspection by TCPHC. Moreover, the RTD detector had not undergone routine inspection and maintenance by the company in at least a year.

The flow diversion valve was linked to the RTD detector used to measure temperature. The company had also failed to conduct routine inspections and maintenance of this valve. In addition, the vent of the surge tank could be exposed to the air outside the tank. In the surge tank, the spray ball could come into contact with milk if the milk overflowed. The milk-filling machine had been operated with inappropriate sanitation for hand hygiene, inadequate transfer of the paper packaging to the storage box, and improper routine maintenance. No health problems linked to the paper packaging material had been reported to the package-processing company. The pipeline between the surge tank and the milk-filling machine had not undergone routine inspection and maintenance. Several of the pipeline connections had an insufficiently shaped and insufficient rubber seal at the joints that permitted the leakage of milk.

Regarding the basic prerequisite program for food safety, company T was found to have inappropriate hand hygiene, including inadequate hand washing and hand sanitizer use before processing and/or during processing. Moreover, boots were insufficiently washed and sanitized.

The processing company did not implement a HACCP plan or have a processing manual and did not have a milk processing record, health check record for staff, or logistic record for milk. Company T used refrigerated trucks for the delivery of milk. On the other hand, the company did not record the temperature of the refrigerator in the trucks. Finally, the refrigerator did not undergo assessment or recording of the temperature every hour.

Analysis of the CCPs generated a score of 20 violations for company T (Table 2). The most important CCPs were the heat-treatment machine, surge tank, and milk-filling machine, which were considered to be the possible sources of contamination. The heat-treatment machine should undergo routine maintenance, and the temperature sensor, temperature record chart, flow diversion valve, and pipeline joints should be fixed to ensure appropriate functioning of the milk processing system. The surge tank should not be exposed to the outside air through the vent, and the spray ball should not be able to contaminate the milk. The milk-filling machine should undergo appropriate maintenance and repair. Finally, employees followed an inappropriate sanitization procedure for packing paper into the machines.

## Discussion

### Summary of the outbreak

Of those who consumed the school lunch, 1,896 people at 25 schools developed gastrointestinal illness. Most drank the school lunch milk during June 14–16, 2021. The milk was supplied by company T, which did not destroy *E. coli* due to inadequate pasteurization. Inspection of the company T revealed hazards associated with the heat-treatment and packaging of the milk.

### Control of the microbiological agent contamination

In company T, the heat-treatment machine used an RTD sensor to measure temperature. This sensor measures temperature based on metal resistance. Although the detector was originally fitted with 20 copper wires, 18 were found to be missing or damaged in the inspection of the facility. Consequently, the RTD might have overestimated temperature due to elevated electrical resistance. Although the heat-treatment equipment had received maintenance, the sensor itself had not undergone maintenance. One possible explanation for the loss of the wires might be time-related degradation. Thus, we recommended that company T routinely inspect and maintain the heat-treatment machine and its measurement items at least once a year.

### Importance of the HACCP plan

Company T had not implemented the HACCP plan, processing manual, and associated processes, even though the Japanese MHLW applied HACCP for food hygiene control to all food processing companies in Japan in June 2021^3^^)^. MHLW announced the implementation of the act in June 2020, and companies should have been able to prepare for the HACCP plan and other processes. Our hazard analysis revealed that the company had insufficient procedures for milk processing, including insufficient washing of the plant, no routine inspection or maintenance of the processing plant, and substandard basic hygiene control for all processing steps. Such procedures may not be related to the size of a company^14^^)^. A previous study of Japanese milk companies determined that the context risk was moderate to high for monitoring and quality assurance activities^15^^)^. We recommended that the facility implement the HACCP plan, processing manual, and records for the milk processing in order to prevent foodborne diseases.

### Source of infection

Our data showed that the milk from company T was the only common item consumed in the 25 schools during this outbreak. However, schools and childcare facilities were supplied by two other milk processing companies. TCPHC identified an unusual number of gastrointestinal illnesses and/or school absences at 25 schools supplied by company T. In the two schools in the menu-based-cohort study, most students consumed school lunch milk on all three days (June 16–18), and symptom onset clustered shortly after this exposure period (**SupplementaryTable 3**). Company T was subsequently confirmed to have supplied the contaminated milk to schools. No other food items were common foods, and the processing manual and record of food processing were sufficient for food processing. Thus, no other foods other than the contaminated milk showed evidence of contamination and were common to the cases. Therefore, the outbreak was judged to have been caused by the school lunch milk supplied by company T.

### Suspected microbiological agent

Our investigation showed that the suspected source of infection was the school lunch consumed during June 14–16. The onset dates and times of cases were during June 15–18. The main symptoms were abdominal pain and diarrhea. Our epidemiological investigation suggested that the incubation period (approximately 2–4 days) and clinical symptoms were compatible with infection by diarrheagenic *E. coli*. However, the *E. coli* isolates obtained from stool and milk samples did not harbor any of the major virulence genes associated with previously characterized diarrheagenic *E. coli* pathogens (e.g., STEC, EPEC, ETEC, EIEC, EAEC, or DAEC) ^13^^)^. Therefore, the causative agent might represent a diarrheagenic *E. coli* lacking currently recognized major pathogenic factor genes, although further investigation would be necessary to clarify its pathogenic potential. Nevertheless, O-genotyping-^16^^)^ detected Gp9 in almost all of stool and milk isolates, suggesting a possible epidemiological link between cases and the suspected source of infection. ^17^^)^ Although causality cannot be definitively established based on the absence of the major virulence factor genes^17^^,^^18^^)^, previous studies ^19^^)^ have reported alternative virulence-associated elements in *E. coli*, such as the type VI secretion system (T6SS)^20^^)^, ETT2, and the capsule-associated gene cluster (e.g. *kps*). Our previous study, using genomic analysis and animal models, suggested that these elements may be critical virulence factors contributing to the pathogenicity of this strain ^20^^)^.

### Pathogenicity for the suspected agent

We found no severe or fatal cases. The main gastrointestinal illnesses reported were abdominal pain and/or diarrhea. Almost all cases were nursery school or elementary school students <20 years old, and no cases occurred in individuals from hospitals and >70 years old who lived in long-term care facilities that were associated with consumption of the same batch of milk from processing company T. We asked a nursing home supplied by the company about the health condition of the residents. Although the residents habitually develop gastrointestinal illness, there was no noticeable increase in incidence on the relevant dates. Susceptibility might be higher in children than in older adults, and we aim to investigate this aspect of the virulence factor of *E. coli* OgGp9.

### Limitations

We were unable to identify the causative agent in this investigation. In addition, our case definition, which included individuals presenting with non-specific symptoms such as abdominal pain and fever as we prioritized sensitivity to ensure comprehensive case capture. It may have introduced some degree of misclassification, particularly among younger children. We were unable to collect stool samples from all students due to the ethical concerns of some of their guardians. Therefore, we conducted a menu-based cohort study from just two schools. However, the number of subjects was insufficient because the milk was consumed by nearly all students, the number of non-consumers was extremely small, resulting in limited statistical power to detect a significant association. There was a significant amount of missing information for students who did not consume it. The relationship between absenteeism and consumption of milk from different milk-processing companies could not be evaluated on an individual basis because the Board of Education is prohibited from sharing personal information.

### Conclusions

The large outbreak was caused by contaminated school lunch milk from company T. The cause of the contaminated milk appears to have been improper sterilization due to inadequate pasteurization or processing. Thus, we recommended the following control measures:

1) The company should routinely inspect and maintain all heat-treatment equipment.

2) The company should implement the HACCP plan, processing manual, and record of processing to prevent hazards.

## Data availability

Data sharing in not applicable to this article as a public health measure based on the food sanitation act.

## Funding statement

This work was supported by a Health and Labour Sciences Research Grant for Research on Food Safety (23KA1005) from the Ministry of Health, Labour and Welfare of Japan.

## Conflict of interest

None declared.

## Ethical statement

The outbreak investigation was based on the Japanese Food Sanitation Act and ethics committee approval was thus not required. We assert that all procedures contributing to this work comply with the ethical standards of the relevant national and institutional committees on human experimentation and with the Helsinki Declaration of 1975, as revised in 2008.

## Patient consent statement

The outbreak investigation was based on the Japanese Food Sanitation Act so we had no require for the patient consent.

## Permission to reproduce material from other source

We had no permission to reproduce material from other sources

## Use of artificial intelligence tools

None declared.

## Authors’ contributions

Katsumi Mizukami managed the epidemiological investigation and performed inspections and microbiological tests. Noriko Noguchi, Kazutaka Nakada, Akina Raijou, and Yuichiro Hyakkoku performed the microbiological tests and analyses. Yuichiro Yahata performed epidemiological and hazard analyses and drafted the manuscript. Yuuki Tsuchihashi performed epidemiological analyses. Kaoru Kurosaki, Hideko Kusunoki, Natsuki Yokota, Mayumi Eino, Takeo Edo, and Tomikatu Suzuki carried out the epidemiological investigation and performed inspections and microbiological tests. Keiko Tsukada performed event-based surveillance and epidemiological analysis. Kenji Takinami supervised the investigation as a public health authority. Yukiko Hara-Kudo supervised the microbiological tests and analyses. Tomimasa Sunagawa supervised the epidemiological investigation and analyses.

Supplementary materials
