# Supplementary material for: A Large Outbreak of Gastrointestinal Infections Associated with Escherichia coli and Consumption of Milk Served in 25 schools in Toyama, Japan, June 2021
Source: Food Saf (Tokyo). 2026 Sep 25;14(3):94–103. doi: 10.14252/foodsafetyfscj.D-25-00027 (PMC13615324; doi:10.14252/foodsafetyfscj.D-25-00027)
Supplement: Supplementary file 1 [file foodsafetyfscj-14-3-94-s001.pdf]

Supplementary materials

Supplemental Table 1. Attack rate among milk consumers, by type of school

Type of school	Number of institutions	Number of cases	Number who consumed milk	Attack rate (%) [a)]
Elementary school	12	1,097	3,336	33%
Junior high school	4	481	1,563	31%
Nursery school	5	173	664	26%
Other school [b)]	4	145	680	21%
Total	25	1,896	6,243	30%

a) Attack rate was calculated as the number of cases divided by the number of individuals who consumed milk. The analysis was primarily based on cross-sectional data across facilities, with retrospective cohort components in two schools.
b) Including special support curriculum schools

Supplemental Table 2. Distribution of symptoms for cases (students and staff) during the gastrointestinal illness outbreak in Toyama involving 25 schools (kindergartens and primary and secondary schools) in June 2021 (N=1,850)

Age group (years)	Number of cases	Abdominal pain		Diarrhea		Nausea		Fever	
		n	(%)	n	(%)	n	(%)	n	(%)
1–4	113	23	(20%)	53	(47%)	21	(19%)	49	(43%)
5–9	802	626	(78%)	386	(48%)	220	(27%)	264	(33%)
10–14	894	786	(88%)	538	(60%)	294	(33%)	191	(21%)
15–19	24	21	(88%)	17	(71%)	8	(33%)	11	(46%)
20–69	17	16	(94%)	12	(71%)	4	(24%)	2	(12%)
Total	1,850	1,472	(80%)	1,006	(54%)	547	(30%)	517	(28%)

Supplemental Table 3. Distribution of school lunch milk consumption frequency among cases and non-cases

	Date of onset				Cases	Non-cases
	June 16	June 17	June 18	June 19		
Number of times school lunch milk was consumed						
Never	0 (0%)	1 (2%)	1 (3%)	0 (0%)	2 (2%)	14 (7%)
Once	0 (0%)	0 (0%)	0 (0%)	0 (0%)	0 (0%)	1 (<1%)
Twice	1 (33%)	0 (0%)	4 (14%)	0 (0%)	5 (6%)	9 (4%)
Three times	2 (67%)	55 (98%)	24 (83%)	1 (100%)	82 (92%)	189 (89%)
Total	3	56	29	1	89	213

We analyzed the relationship between the number of times school milk was consumed and the development of gastrointestinal illness. Overall, 82 of the cases (92%) consumed the school lunch milk three times while 189 of the non-cases did so (89%). The highest number of cases of onset was on June 17 (55 cases).

Supplemental Table 4. Proportion of milk consumed and common school lunch foods in June 14–16 in Elementary school 1 and Elementary school 2

		Consumed	/Total	(%)
June 14, 2021 (Miso soup)				
Elementary school 1	Milk [a)]	189	/204	(93%)
	Miso soup [b)]	202	/206	(98%)
Elementary school 2	Milk	91	/93	(98%)
	Miso soup	Not	served	—
June 15, 2021 (Green salad)				
Elementary school 1	Milk [b)]	191	/206	(93%)
	Green salad [c)]	194	/205	(95%)
Elementary school 2	Milk	92	/93	(99%)
	Green salad	93	/93	(100%)
June 16, 2021 (Carrot soy sauce pickle)				
Elementary school 1	Milk [b)]	187	/206	(91%)
	Carrot soy sauce pickle [a)]	194	/204	(95%)
Elementary school 2	Milk	89	/93	(96%)
	Carrot soy sauce pickle	Not	served	—

a) except for four cases with missing information on food consumed
b) except for two cases with missing information on food consumed
c) except for three cases with missing information on food consumed
No other common foods

Supplemental Table 5. Annual regular inspection program

1. Overview of the facility
    - (1) Number of employees (including part-time jobs)
    - (2) Working day and time
    - (3) Water supply system (objective of water use, well water)
    - (4) Management of water tank
    - (5) Use of sewer system
    - (6) Food safety supervisor
    - (7) Rodent and pest control
2. Confirmation of products
    - (1) Main processing products and volume of products
    - (2) Main client of the company and trading area
3. Health check of employee(s)
    - (1) Fecal sample test for food handlers every month (*Shigella*, *Salmonella*, enterohemorrhagic *Escherichia coli* O157/2/O111)
    - (2) Hygiene education of employee(s)
    - (3) Record of health check for employee(s)
    - (4) Condition and location of han washing machine
    - (5) Implementation of a standardized operation manual
4. Processing of products
    - (1) Ingredients
        - 1) Record of the condition of received maerials
        - 2) Condition of preserving maerials
    - (2) Record of temperature of refrigerator
    - (3) Processing of products
    - (4) Use of metal detector
    - (5) Storing of inspection of the food sample
        - 1) Preserving period
        - 2) Preserving method
        - 3) Lot management
    - (6) Test of standard criteria for milk
    - (7) Evidence of use-by date based on the Food Sanitation Act
5. Others
    - (1) Complaints about products and reuse of products
    - (2) Condition of facility

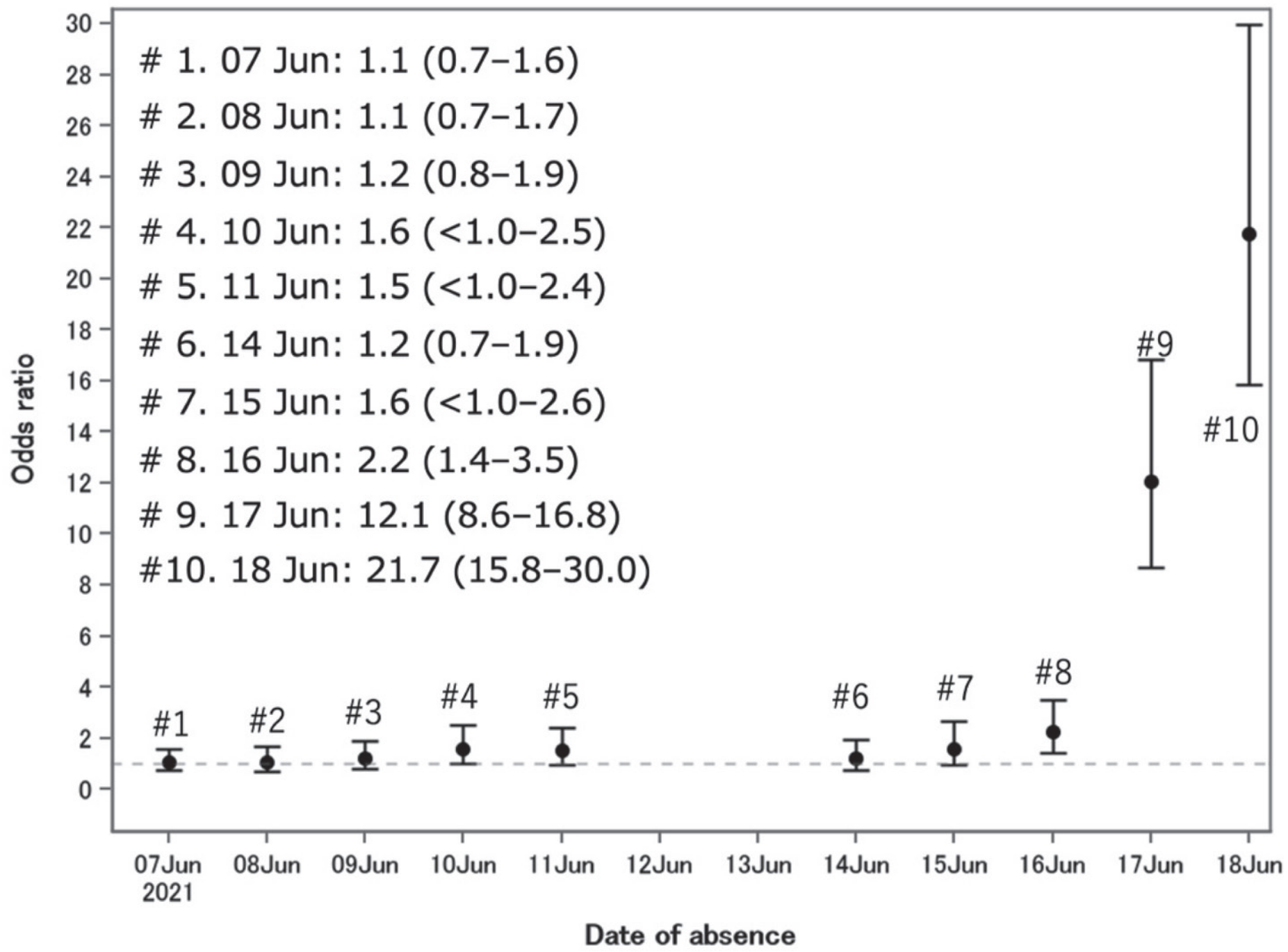

Supplemental Figure 1. Odds ratios for absence or early departure by school milk supplier, Toyama, June 2021
Odds ratios compare students enrolled in schools that received milk from company T with students enrolled in schools that received milk from other processing companies. The outcome was absence from school or early departure on each date. Labels #1–#10 correspond to the estimates listed in the inset.
